# Retraction: Gender-specific impact of stress and adiposity on autonomic stress modulation in teachers

**DOI:** 10.3389/fpsyg.2026.1930567

**Published:** 2026-07-13

**Authors:** Frontiers Editorial Office

The journal retracts the 01 May 2025 article cited above.

Following publication, concerns were raised about the potential publication of duplicate content in the article cited above and the following article published in Frontiers in Psychology: https://www.frontiersin.org/journals/psychology/articles/10.3389/fpsyg.2024.1422709/full.

An investigation was conducted in accordance with Frontiers' policies, whereby it was confirmed that there is a substantial overlap and minimal new information compared to the previously published work. Therefore, following the Committee on Publication Ethics guidelines, this article, published more recently, is deemed to be redundant and as such is retracted.

This retraction was approved by the Chief Editors of Frontiers in Psychology and the Chief Executive Editor of Frontiers. The authors have not responded to correspondence regarding this retraction.

